# Effect of reminders on mitigating participation bias in a case-control study

**DOI:** 10.1186/1471-2288-11-33

**Published:** 2011-03-31

**Authors:** Clarence C Tam, Craig D Higgins, Laura C Rodrigues

**Affiliations:** 1 Faculty of Epidemiology and Population Health, London School of Hygiene & Tropical Medicine, London, UK

**Keywords:** bias, case-control studies, epidemiologic methods, survey methods, data collection, nonrespondents, respondents, community surveys

## Abstract

**Background:**

Researchers commonly employ strategies to increase participation in health studies. These include use of incentives and intensive reminders. There is, however, little evidence regarding the quantitative effect that such strategies have on study results. We present an analysis of data from a case-control study of *Campylobacter *enteritis in England to assess the usefulness of a two-reminder strategy for control recruitment.

**Methods:**

We compared sociodemographic characteristics of participants and non-participants, and calculated odds ratio estimates for a wide range of risk factors by mailing wave.

**Results:**

Non-participants were more often male, younger and from more deprived areas. Among participants, early responders were more likely to be female, older and live in less deprived areas, but despite these differences, we found little evidence of a systematic bias in the results when using data from early reponders only.

**Conclusions:**

We conclude that the main benefit of using reminders in our study was the gain in statistical power from a larger sample size.

## Background

The selective inclusion in health studies of individuals whose participation is dependent on the outcome and risk factors being investigated is a common problem in epidemiology. Case-control studies, in which exposure information is collected after diagnosis of the outcome, are particularly susceptible to such bias. Individuals suffering from the condition being investigated are likely to be more interested in participating, particularly if they are aware of, and are exposed to, risk factors for the condition. Conversely, healthy individuals in the population may be less likely to participate, and participation is often related to other factors that may be correlated with exposure, such as age, gender, socioeconomic position and educational level [1,2]. Correction for such bias in the analysis is not possible without relevant information on non-participants, which is usually unavailable.

A commonly recommended way to minimize bias from non-participation is to increase participation, in the hope of minimizing systematic differences between participants and non-participants. A number of strategies are employed by researchers for this purpose, including use of different modes of contact, incentives, reminders, shorter data collection instruments, and more engaging documentation. In a review and meta-regression of 26 case-control studies conducted over 13 years in Germany, Stang et al. found that studies using multiple contact modes (e.g. mail and telephone) had higher participation than those using letters as the only form of contact [3]. In a recent systematic review of randomized trials of participation in postal and electronic surveys not restricted to health studies, Edwards et al. identified a number of factors for which demonstrable evidence existed of an effect on participation rates. Use of (particularly monetary) incentives included with the questionnaire, shorter and more interesting questionnaires, recorded delivery, prior contact, follow-up contact, providing a second copy of the questionnaire with the follow-up, personalized questionnaires, handwritten addresses on the envelopes, use of stamped return envelopes and university affiliation were all associated with increases in participation [4]. In another review by Nakash et al. that focused on trials of methods to increase response to postal questionnaires pertaining to health research it was found that strategies using intensive reminders and shorter questionnaires resulted in higher participation. Incentives did not increase participation, although that review included only studies recruiting patients receiving treatment, who may already have had a high incentive to participate [5].

Despite the evidence that these strategies can improve participation, much less evidence exists about the quantitative effect that increased participation rates have on study results. Stang et al. demonstrated in simulations that studies with lower participation rates can in some situations result in less bias [6]. This might occur if late responders in studies with higher participation have a higher probability of non-differential misclassification. In a case-control study, such non-differential misclassification would occur if late responders reported exposure to risk factors less accurately than early responders - for example, because of poorer recall - but the degree to which they mis-reported exposure was no different among those who were ill (cases) and those who were not (controls). The effect of strategies to increase participation on study results is thus difficult to predict, and will depend on numerous factors, including the type of study design, the question being investigated, the invasiveness of the data collection process, and the period of time over which information needs to be collected. In the context of case-control studies, evidence of the impact of such strategies is limited and conflicting. In a study of renal cell carcinoma, Kreiger et al. found that follow-up intensity had little effect on the effect estimates [7]; in another study of breast cancer using a validation substudy to assess the accuracy of recall on use of antihypertensive drugs, Voigt et al. found that information provided by late responders was no less accurate than that provided by early responders, but analyzing data from early responders only resulted in considerable bias [8].

In this paper, we present an analysis of the effect of mail reminders to increase participation among controls in a case-control study of risk factors for *Campylobacter *enteritis in England.

## Methods

Between April 2005 and June 2006, we conducted a case-control study of risk factors for *Campylobacter *enteritis among individuals aged 18 years and above in five Health Protection Units (HPU) in England. The details of the study are extensively described elsewhere [9]. Laboratory-confirmed cases of *Campylobacter *enteritis reported within each HPU were sent a letter from the local Consultant in Communicable Disease Control (CCDC) inviting them to participate in the study, together with a consent form, a 12-page, self-administered risk factor questionnaire, and a pre-paid, addressed return envelope. The questionnaire enquired about health details (presence of diabetes and chronic gastrointestinal illness, and use of acid-suppressing medications), exposure to animals in the home, workplace or elsewhere, recreational exposure to water sources, and a detailed history of normal dietary habits as well as consumption of chicken, and untreated dairy and water in the five days prior to illness onset. No reminders were sent to cases, as a pilot study indicated that there was little benefit in doing so.

Controls were randomly sampled from lists of individuals registered with general practice clinics in the five HPUs. Based on previous years' distribution of reported cases, five times as many controls as expected cases were sampled in each HPU, frequency matched on age group, sex and month of report. Potential controls were approached with an initial mailing pack similar to that used for cases. Individuals who had not responded within two weeks were sent a reminder letter. A second reminder and another copy of the questionnaire were sent to those who had still not responded after three weeks. Controls were asked for the same risk factor information as cases, but for recent risk factors we sought information about exposure in the five days prior to questionnaire completion.

The study received a favorable ethical opinion from the North West Multicentre Research Ethics Committee. Approval was obtained from Local Research Management and Governance departments serving each study site.

Overall participation was 46.5% (n = 2381) among cases and 37.3% (n = 5256) among controls. In the original study, we excluded individuals reporting irritable bowel syndrome (cases = 221, 9.3%; controls = 324, 6.2%), because of difficulties ascertaining date of onset and because risk factors in this group may differ. We additionally excluded controls reporting gastrointestinal symptoms in the previous 14 days (n = 431, 8.2%), and cases and controls reporting foreign travel in the 14 days prior to illness onset or questionnaire completion, respectively (cases = 560, 23.5%; controls = 511, 9.7%). Finally, we excluded two cases and seven controls because we could not determine whether they were aged 18 years or above, and a further six cases that occurred in the same household as a previously identified case. After exclusions, 1592 and 3983 cases and controls were available for analysis. In the final multivariable model, self-reported, past *Campylobacter *enteritis, use of acid-suppressing medications, recent acquisition of a pet dog, and consumption of chicken prepared outside the home were identified as risk factors for *Campylobacter *enteritis.

In our previous analysis [9], the potential for bias due to non-participation was assessed using inverse probability weighting, where the weights were inversely proportional to the probability of participation and derived from a two-level logistic model regressing participation against study site, a three-way interaction between age group, sex and case/control status, and area of residence as a latent, random intercept variable capturing area-level deprivation. The analysis indicated that weighting made little difference to the effect estimates for risk factors identified in the final multivariable model.

For the present analysis, we categorized controls as follows: (1) individuals who returned a completed questionnaire and were included in the anaysis (included controls); (2) individuals who returned a completed questionnaire and were subsequently excluded from analysis because of the above-mentioned reasons (excluded controls); (3) individuals who declined to participate (active refusers); (4) inviduals sent a questionnaire but whose address details were subsequently found to be incorrect or invalid (incorrect addresses); and (5) individuals from whom no response was obtained after three reminders (passive refusers). Included controls (group 1) were further categorized as (A) controls who completed or returned a questionnaire within two weeks of the initial contact; (B) controls who completed or returned a questionnaire after being sent the first reminder, but before a second reminder was sent out; and (C) controls who returned a questionnaire after being sent a second reminder.

We compared controls in groups 1 to 5 with respect to the distribution of age group, sex and area-level deprivation. We obtained the latter by linking individuals' postcodes of residence to Super Output Areas (SOAs), geographical boundaries comprising approximately 1000 residents for which aggregated census data are available. SOAs are ranked according to a standard Index of Multiple Deprivation (IMD) [10], which captures geographic variation in deprivation, using a range of education, employment, health, crime, housing and environment indicators. Individuals were assigned to a quintile of deprivation based on their SOA of residence. The distributions of these variables between the five groups were tabulated. For a small fraction of individuals, HPUs were unable to provide information on age (n = 344, 2.3%), sex (n = 66, 0.4%), or postcode (133, n = 0.9%). These individuals were excluded from analysis of the relevant variable, but included in other comparisons for which data were available.

For included controls (group 1), we investigated the effect of each wave of reminders on mitigating participation bias by estimating the effect of individual risk factors on case status for those returning a questionnaire before the first reminder (group 1A), those returning a questionnaire before the second reminder (1A+1B) and all included controls (1A+1B+1C). We used unconditional logistic regression adjusting for the stratifying variables of age group, sex, study site and month. For each risk factor, we calculated the absolute difference in the effect estimate, *δ*, as the difference in the regression coefficient between group 1A and all controls, and groups 1A+1B and all controls:

where *β_i,all _*represents the logarithm of the odds ratio for risk factor *i *using all controls, and *β_i,j _*is the logarithm of the OR for risk factor *i *using controls *j *(*j *= 1A, 1A+1B). For each mailing wave, we determined the proportion of variables yielding Wald test p-values < 0.2, according to the conventional practice of selecting such variables for further analysis in a stepwise regression.

Even in the absence of systematic error, differences in the coefficients occur due to random error. The extent of this error is dependent on the prevalence of the risk factor, as for a given sample size random error increases with decreasing prevalence. To assess whether bias might have occurred that exceeded that expected from random error, we plotted absolute bias against prevalence for each risk factor, by mailing wave.

In addition, we investigated the effect on the final multivariable model of using only initial respondents, and participants responding before a second reminder, as compared with the analysis using all controls.

Analysis was performed using Stata 10 (Stata Corporation, Texas) and Microsoft Excel 2007 (Microsoft Corporation, Washington) software.

## Results

The distribution of sex, age group and area-level deprivation for each of the five groups is shown in Table 1. Compared with all sampled potential controls, participants (groups 1 and 2) had a greater proportion of females and tended to reside in less deprived areas. Excluded controls also had a greater proportion of middle-aged individuals. Among active refusers, over a third were over 65 years of age. By contrast, passive refusers were more likely to be male, younger and reside in areas of greater deprivation. Similarly, among those with incorrectly-recorded addresses, two-thirds were male, aged 18 to 44 years, and resided in areas in the lowest three quintiles of deprivation.

**Table 1:** Distribution of Demographic and Socioeconomic Variables Among Included, Excluded and Potential Controls in a Case-Control Study of *Campylobacter *Enteritis, England 2005-6

	Group	1	2	3	4	5		
		**Included controls**	**Excluded controls**	**Active refusers**	**Incorrect addresses**	**Passive refusers**	***All***	***%***

Sex	Female	54.0%	58.1%	50.7%	33.6%	43.1%	*7,111*	*48.0%*
	Male	46.0%	41.9%	49.3%	66.4%	56.9%	*7,703*	*52.0%*
	***Total^§^***	***3,983***	***1,283***	***2,200***	***749***	***6,599***	***14,814***	
								
Age group	18-24	8.9%	6.8%	7.2%	21.8%	16.9%	*1,728*	*11.9%*
	25-34	17.9%	14.7%	11.5%	24.4%	23.4%	*2,628*	*18.1%*
	35-44	19.6%	18.8%	14.8%	21.7%	22.6%	*2,992*	*20.6%*
	45-54	19.6%	24.7%	14.0%	12.7%	15.6%	*2,506*	*17.2%*
	55-64	14.3%	17.1%	15.1%	8.7%	10.1%	*2,009*	*13.8%*
	65+	19.6%	17.8%	37.4%	10.8%	11.3%	*2,673*	*18.4%*
	***Total^†^***	***3,983***	***1,181***	***2,144***	***743***	***6,485***	***14,536***	
								
IMD quintile	1 (most deprived)	16.5%	15.4%	19.3%	26.4%	27.3%	*3,267*	*22.2%*
	2	16.2%	16.4%	19.7%	20.2%	19.6%	*2,727*	*18.5%*
	3	19.9%	19.3%	18.4%	19.2%	18.7%	*2,808*	*19.0%*
	4	21.5%	22.2%	20.0%	15.5%	17.5%	*2,837*	*19.2%*
	5	25.8%	26.7%	22.6%	18.7%	16.9%	*3,106*	*21.1%*
	***Total^‡^***	***3,912***	***1,283***	***2,200***	***749***	***6,601***	***14,745***	

^§ ^Sex was not known for 66 individuals

^† ^Age was not known for 344 individuals

^‡ ^IMD quintile was missing for 135 individuals

Among included controls, groups 1A and 1B were similar in terms of age distribution, age at leaving full-time education and area-level deprivation, but controls returning a questionnaire before the first reminder were more likely to be female. In contrast, individuals returning a questionnaire after a second reminder were more likely to be younger, have left full-time education at age 16 years or still be in full-time education, and reside in an area of greater deprivation (Table 2).

**Table 2:** Distribution of Demographic and Socioeconomic Variables Among Included Controls by Mailing Wave in a Case-Control Study of *Campylobacter *Enteritis, England 2005-6

	Group	1A	1B	1C	
		**First mailing**	**First reminder**	**Second reminder**	**Total*****^§^***

Sex	Female	56.0%	49.5%	51.9%	53.3%
*χ^2 ^p = 0.004*	Male	44.0%	50.5%	48.1%	45.2%
	Total	2507	703	717	3927
					
Age group	18-24	5.4%	6.0%	10.3%	6.3%
*χ^2 ^p < 0.001*	25-34	12.6%	13.1%	14.8%	12.9%
	35-44	19.0%	20.5%	24.7%	20.0%
	45-54	20.0%	21.2%	20.8%	20.1%
	55-64	20.0%	19.2%	15.1%	18.7%
	65+	23.1%	20.1%	14.4%	20.7%
	***Total***	***2507***	***703***	***717***	***3927***
					
Age at leaving full-time education	< 16 yrs	28.3%	30.9%	26.1%	28.3%
*χ^2 ^p = 0.007*	16 yrs	29.4%	29.3%	34.6%	30.4%
	17 yrs	9.0%	9.5%	8.8%	9.0%
	18 yrs	11.5%	9.1%	10.3%	10.9%
	19 yrs	20.5%	19.2%	17.1%	19.6%
	Still in education	1.3%	1.9%	3.1%	1.8%
	***Total^†^***	***2463***	***692***	***706***	***3861***
					
IMD quintile	1 (most deprived)	15.2%	18.4%	19.0%	16.5%
*χ^2 ^p = 0.056*	2	16.0%	14.8%	18.6%	16.2%
	3	20.3%	18.8%	19.0%	19.8%
	4	22.5%	21.2%	19.0%	21.6%
	5	26.0%	26.8%	24.4%	25.9%
	***Total^‡^***	***2483***	***697***	***710***	***3890***

^§ ^56 individuals could not be assigned to any group due to missing date information

^† ^Age at leaving full-time education was missing for 66 individuals

^‡ ^IMD quintile was missing for 71 individuals

In total, 110 indicator variables were tested in the regression models. Of these, 58 yielded Wald test p-values < 0.2 after adjustment for sex, age group, study site and month regardless of reminder wave. A further four variables were selected using this criterion when using all controls only, while another four were selected when using groups 1A or 1A+1B, but not when all controls were included. However, all of these latter eight variables had p-values close to 0.2 and 80% confidence intervals (CIs) for the OR close to one.

**Figure 1:**
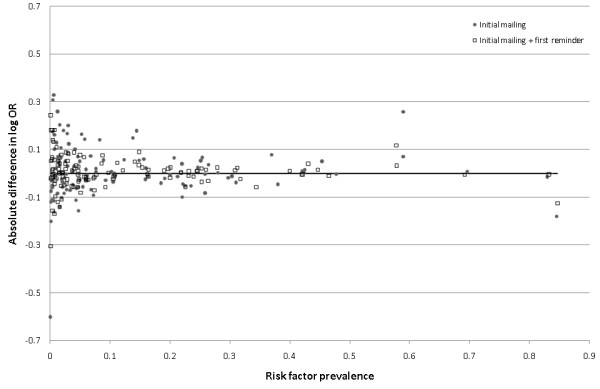
**Absolute difference in log OR comparing models using all controls with models using controls responding before first and second reminders, against risk factor prevalence**. Each point represents the absolute difference in the log OR for a potential risk factor compared with an analysis using all available controls. Closed circles: models using controls returning a questionnaire before being sent a reminder; Open squares: models using controls returning a questionnaire before being sent a second reminder. All models are additionally adjusted for sex, age group, study site and month.

Figure 1 shows, by mailing wave, the absolute difference in the log OR of all variables relative to an analysis using all controls, plotted against the unadjusted prevalence of each variable. The differences are clearly centered around zero, and decrease in magnitude with increasing prevalence and mailing wave, indicating that greater random error resulting from lower risk factor prevalence or smaller sample size were primarily responsible for observed differences in the log OR.

**Figure 2:**
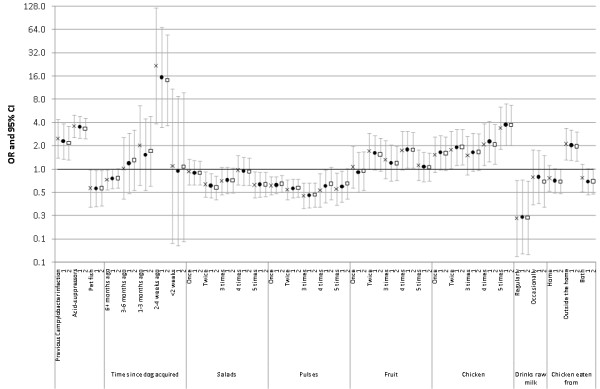
**ORs and 95% CIs for *Campylobacter *risk factors in final multivariable model, by mailing round**. Crosses: model using controls returning a questionnaire before being sent a reminder; Closed circles: model using controls returning a questionnaire before being sent a second reminder; Open squares: model using all controls. All models are additionally adjusted for sex, age group, study site and month.

Figure 2 shows, by mailing wave, ORs and 95% CIs for all factors included in the final multivariable model, adjusted for sex, age group, study site and month. For all variables, there are only marginal differences in the ORs, and the 95% CIs include the point estimates from the other models using alternative groups of controls.

## Discussion

Our analysis has shown that, in our study, use of additional reminders among controls had little effect on mitigating bias due to non-participation. Despite some differences between early and late responders in terms of sex, age, educational level and area-level deprivation, using only data from early responders resulted in small differences in the effect estimates relative to using all controls. These differences were mainly due to random error and were minor in comparison to the uncertainty in the estimates. The main benefit of using reminders in our study was thus the gain in statistical power resulting from the larger sample size.

In this analysis we are unable to assess the effect of true participation bias, that is, the potential from bias resulting from systematic differences between participants and non-participants, on whom limited information was available. In the original study, we adjusted for differences between participants and non-participants in terms of age, sex, study site and area-level deprivation, and concluded that these factors made little difference to the results [9]. Bias could still have occurred, however, if within strata of these factors, important differences existed between participants and non-participants with respect to other factors related to *Campylobacter *enteritis.

Among non-participants, we also observed important differences between active and passive refusers. Compared with individuals who actively refuse to participate, those from whom no response is obtained are more likely to be male, younger and to live in more deprived areas. A high proportion of active refusers were over 65 years of age. A possible explanation for this is that many of these individuals were in long-term care or had other health conditions that precluded participation. These differences suggest that, when refusal is high, replacement through more intense recruitment from passive refusers may not be adequate to mitigate bias if active refusal is related to factors associated with the outcome of interest.

A number of potential controls approached were subsequently found to have incorrectly recorded or out-of-date addresses. These individuals tended to be male, younger, and lived in more deprived areas. The most likely reason for the incorrect recording of addresses is list inflation, which results from a delay in removing from general practice registers records of individuals who are deceased or no longer living in the area. Studies in the late 1990s estimated that approximately 10% of addresses on general practice registers were incorrect [11], although this figure is believed to have decreased due to recent efforts to reduce list inflation across the National Health Service. Incorrect addresses were ascertained when questionnaires were returned undelivered. This number is probably an underestimate, as it is likely that additional undelivered questionnaires were not returned to us. We know of only two studies that have investigated the fate of incorrectly addressed letters. Sandler et al. found that all envelopes sent to invalid (non-existent) addresses were returned. By contrast, among letters sent to fictitious individuals at valid addresses, 13% were not returned [12]. In Germany, Schmidt-Pokrzywniak et al. found that around 2% of such letters were not returned [13]. We thus expect that a fraction of passive refusers were individuals for whom address details were incorrect, but if the above findings are applicable to our study setting, this fraction should be small.

The presence of participation bias is likely to depend on numerous factors, including some over which researchers have little control. These might include media interest and public awareness in the subject and hypotheses under investigation, and health behaviors that may be related both to participation and risk factors being studied. Even in the absence of systematic differences between participants and non-participants, bias may occur if information from late responders is less accurate than that from early responders. We think this unlikely in our study, as all controls were asked to provide information about exposure to risk factors in the previous five days, regardless of when they completed the questionnaire.

Our findings may be difficult to generalize to other settings, because the effect of response propensity and timeliness may differ depending on the research question and risk factors of interest. Frameworks for addressing participation bias include the Leverage-Saliency Theory put forward by Groves [14]. The theory postulates that, for a given research topic, there is a pool of individuals in the population with a propensity to participate. The likelihood that an individual will participate depends on this so-called leverage, and the saliency with which the topic is presented to them by researchers. The theory predicts that for individuals with a low interest in the topic, other incentives, such as monetary remuneration, can improve participation. Such predictions have been tested using population subgroups for which easily identifiable proxies exist; for example, by gauging teachers' interest in educational surveys [15]. For many health topics, however, these subgroups will be difficult to identify, as interest will only be tangentially related to easily identifiable characteristics such as age and occupation. This will nevertheless be an area of growing importance in epidemiology. It is known that the effort required to achieve comparable participation levels among controls in health studies is increasing over time [16], making assessments of the potential for participation bias increasingly important. Our study indicates that among those with a propensity to participate, there is a fraction for which obtaining a response requires more effort. However, the added benefit in doing so appears to be limited, because these late-responding individuals are not substantively different from early responders in terms of factors relevant to the analysis. Further, if these late responders differ in important ways from passive refusers, then the added effort in recruiting them will be fruitless, because the impact on mitigating bias will be minimal. Instead, focusing additional resources on strategies to engage groups known to have low participation should be more productive. Specific recruitment strategies and study documentation may need to be designed so as to attract in particular young males and those living in more deprived areas.

## Conclusions

In our study, controls who responded early differed from late responders in terms of demographic and socioeconomic factors, but these differences did not influence the results of our risk factor analysis. Pursuing initial non-responders through reminders in case-control studies may thus not be sufficient to mitigate bias if those with a propensity to participate differ in important ways from those who would not participate regardless of how many reminders were sent. Instead, enhancing individuals' propensity to participate in research studies through targeted strategies and study materials aimed at population subgroups with low participation should be more successful at reducing the potential for participation bias.

## Competing interests

The authors declare that they have no competing interests.

## Authors' contributions

CCT and CDH conceived the idea for the study. CCT conducted the analysis. All authors contributed to the interpretation of results and drafting of the manuscript.

## Funding

This study was funded by the United Kingdom Food Standards Agency (Project Number B14011).

## Pre-publication history

The pre-publication history for this paper can be accessed here:

http://www.biomedcentral.com/1471-2288/11/33/prepub
